# Anions as catalysts: from organosilanes as pro-nucleophiles to base-embedding electrophiles

**DOI:** 10.1039/d6cs00804f

**Published:** 2026-11-02

**Authors:** Alireza Abbasi Kejani, Allya Larroza, Arnaldo X. Torres-Hernandez, Paulo H. S. Paioti

**Affiliations:** a Institute of Organic Chemistry and Biochemistry of the Czech Academy of Sciences Prague 160 00 Czech Republic paulo.paioti@uochb.cas.cz; b Department of Organic Chemistry, Faculty of Science, Charles University Prague 128 40 Czech Republic

## Abstract

The catalytic activity of neutral and anionic Lewis base catalysts depends on several factors. One is catalyst turnover, which may explain why neutral Lewis bases, such as tertiary amines and phosphines, are widely employed as catalysts in organic synthesis. In this review, we discuss less common strategies by which fluorides, alkoxides, amide anions and related anionic species can be rendered efficient catalysts, including for stereoselective synthesis. We give historical context and highlight recent examples that exploit organosilanes as pro-nucleophiles for challenging bond formation and enantioselective synthesis. The primary emphasis, however, is placed on the use of organosilanes as pro-bases, specifically referred to herein as base-embedding electrophiles (BEE), as part of an idea we wish to conceptualize, as we believe the concept holds substantial yet underappreciated potential in organic synthesis. We focus on how the BEE reagents disentangle traditional Brønsted acid–base considerations from catalyst turnover in substitutions and additions. Existing examples are discussed with particular attention to their conceptual significance. Drawing on these principles, we and others have leveraged base-embedding electrophiles to develop catalytic and stereoselective reactions toward difficult-to-access organic molecules. By sharing this perspective, we hope that readers will also be able to recognize the opportunities and challenges associated with anionic catalysis enabled by organosilanes.

## Abstract 2

Anions are efficient catalysts when combined with organosilanes as pro-nucleophiles or with base-embedding electrophiles, the latter is an emerging catalytic deprotonation strategy which disentangles turnover from acidity–basicity constraints.

## Graphical abstract

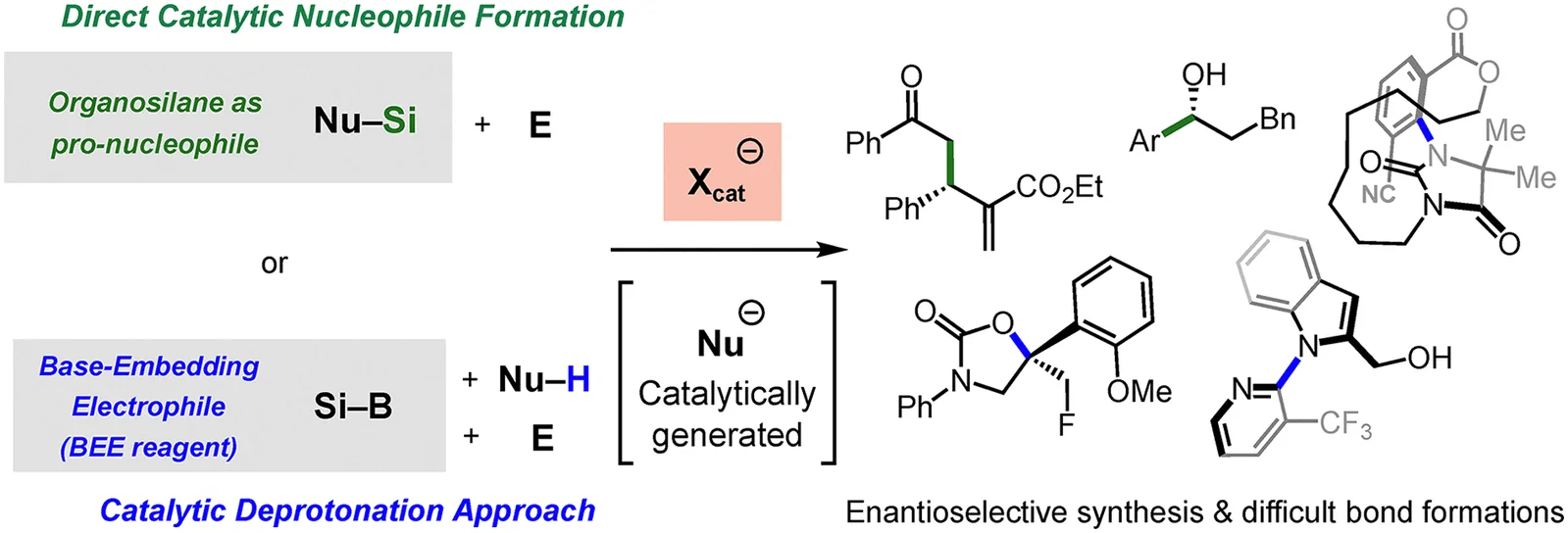

## 1. Introduction

Organosilanes are widely used as synthetic intermediates during preparation of biologically active molecules,^1^ including pharmaceuticals and agrochemicals.^2^ Unlike carbon, silicon can form five or six bonds, a feature leading to several distinct activation modes.^3^ The equilibrium between tetravalent and pentavalent silicon species is especially relevant to chemistry^4^ because it allows a Lewis acid or weaker Lewis base (tetravalent silicon) to be converted to stronger Lewis or Brønsted base (pentavalent) intermediates.^5^ Such activation, broadly defined, has led to myriad well-known and useful organic reactions such as Mukaiyama aldol reaction,^6^ Hiyama–Denmark coupling,^7^ Hosomi–Sakurai allylation^8^ Fleming–Tamao oxidation^9^ and Peterson olefination,^10^ to name a few.

In this review, we discuss catalytic reactions that proceed *via* activation of tetravalent silicon by anionic catalysts which have been disclosed in the past ten years, the exceptions being two historical examples and work which is directly related and helps us to explain the ones presented herein. We start with cases where organosilanes serve as pro-nucleophiles, the most traditional and well-explored reactivity. In 2008, in their seminal review,^11^ Denmark and Beutner conceptualized the use of Lewis bases as catalysts in organic synthesis, where the use of anions as catalysts in combination with pro-nucleophiles, and mechanistic intricacies, were discussed in detail. Here, we present recent enantioselective reactions, including development of new catalysts as well as activation of new bonds. We then move to much less explored classes of reactions where organosilanes act as base-embedding electrophiles (BEEs), which have not been conceptualized previously. We introduce the term BEE for several reasons, the most important being to highlight the uniqueness and potential utility of catalyst turnover, in which an anionic Lewis base reacts with an electrophilic site to generate a Brønsted base. Firstly, not all pro-bases possess electrophilic groups;^12^ thus a BEE is a specific type of a pro-base, one that is not generated by the loss of a proton. Herein, the electrophilic atom of a BEE is silicon in all cases, but other atoms can be envisioned, as long as a species acting as a Brønsted base is released through nucleophilic attack on the electrophilic component of the reagent. In our view, and as will be discussed in more detail later, the term also brings attention to the key step of the catalytic cycles, namely catalyst turnover during which Brønsted base (re)generation takes place. Lastly, and more casually, we favor the term BEE by analogy with bees, invisible pollinators of nature:^13^ BEE reagents are promoters performing a somewhat invisible job, as they often do not end up in the desired product (substitution reactions) or are part of the product yet employed mostly as protecting groups or cleaved upon workup (addition reactions). It is important to distinguish BEEs from latent pro-nucleophiles,^14^ proposed by Vilotijevic and coworkers as part of another emerging and related concept. Latent pro-nucleophiles are species which, by activation with anions, give rise to active nucleophiles catalytically. A BEE gives rise to a Brønsted base instead.

We wish to explain why we became interested in the use of organosilanes as promoters in reactions with anionic Lewis base catalysts. However, prior to that, we wish to give credit to giants in the field who came before us. Remarkably, the first examples of such catalytic reactions have been published independently by Heathcock^15^ as well as by Noyori and Kuwajima^16^ in 1977, in the context of aldol reactions (Fig. 1).

**Fig. 1:**
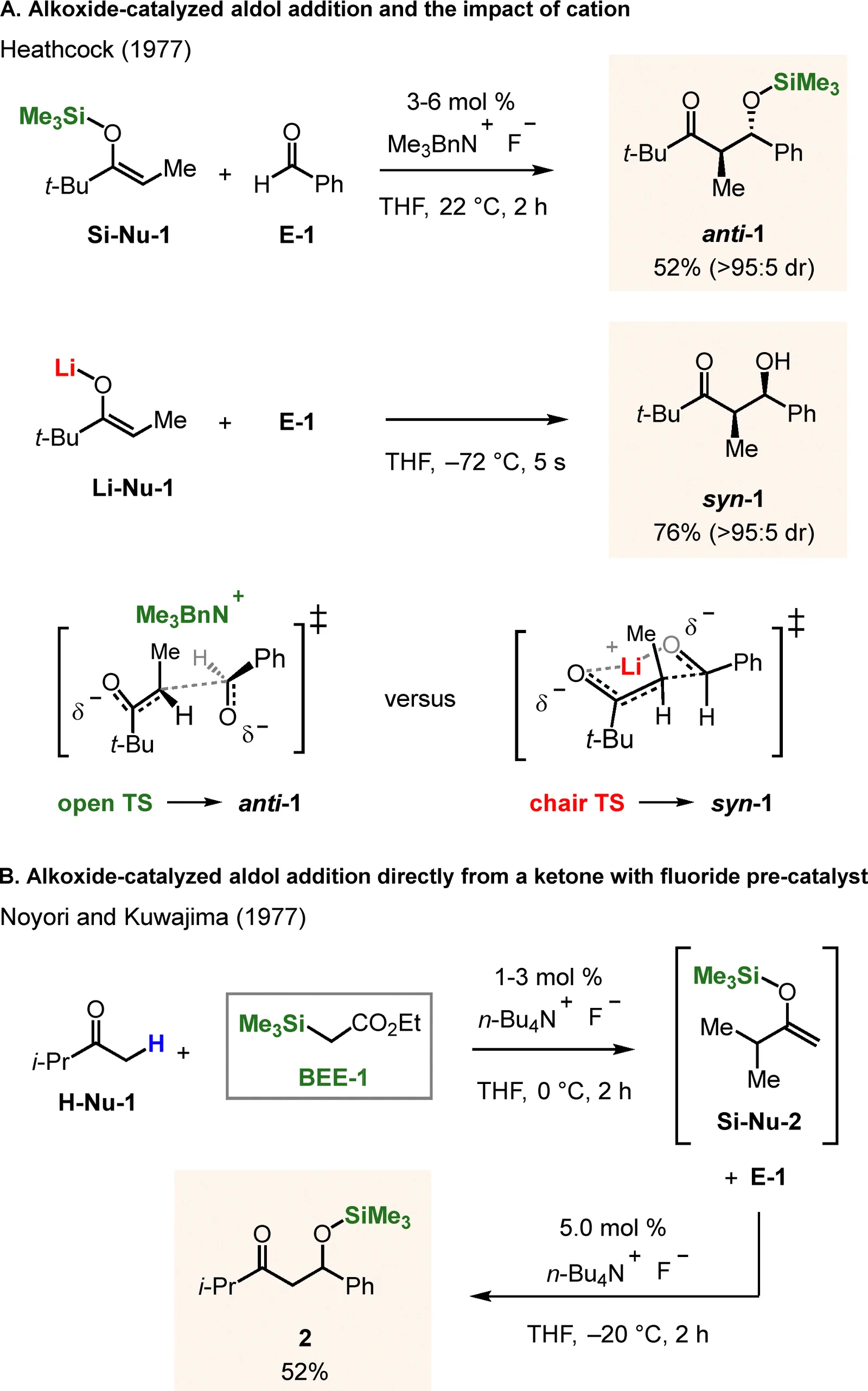
Alkoxide-catalyzed reactions introduced in the context of aldol reactions. Note that the fluoride source serves as the pre-catalyst. (A) The outcome of an aldol reaction with a silyl enol ether *versus* a lithium enolate. (B) *In situ* catalytic preparation of silyl enol ether from a ketone followed by catalytic aldol.

Heathcock and coworkers^15^ disclosed that the silyl enol ether Si-Nu-1 reacts with benzaldehyde in the presence of catalytic trimethylbenzylammonium fluoride to produce the silyl ether *anti*-1 in high diastereoselectivity, at room temperature in two hours (Fig. 1(A)). For the reaction to proceed catalytically, the resulting alkoxide formed by aldol needs to react with the silicon-containing pro-nucleophile (Si-Nu-1). The analogous lithium enolate Li-Nu-1 was reacted at −72 °C, under otherwise identical conditions, to give *syn*-1 with high diastereoselectivity. The impact of the cation is evident. An open transition state toward the *anti* product is favored for the organic cation, whereas a Zimmerman–Traxler^17^ transition state leading to the *syn* product is operative with the lithium-based enolate. The Kuwajima group developed a catalytic silylation of ketones with fluoride as the pre-catalyst.^18^ Later, Kuwajima together with Noyori and coworkers developed a catalytic aldol reaction starting directly from the ketone (H-Nu-1), thus bypassing the need for pre-forming the silyl enol ether (Fig. 1(B)).^16^ The protocol consists of *in situ* catalytic preparation of silyl enol ether Si-Nu-2 involving ethyl trimethylsilylacetate (BEE-1), followed by a catalytic aldol promoted by tetrabutylammonium fluoride, in much the same way as that reported by Heathcock. BEE-1 reacts with fluoride to generate, catalytically, an enolate that functions as the base which deprotonates the ketone.

We recently developed intermolecular nucleophilic aromatic substitution (S_N_Ar) reactions for synthesis of a broad range of racemic, diverse and diversifiable (hetero)biaryl atropisomers with an N–C stereogenic axis.^19^ The reactions were promoted using 2.0 equiv. Cs_2_CO_3_ in reagent grade DMSO as the solvent. The efficiency and practicality are ideal for medicinal chemistry screening. While contemplating strategies for eventually developing an enantioselective (atroposelective) S_N_Ar variant, we anticipated major challenges regarding both selectivity and reactivity. We were aware that (1) achieving high levels of enantio-differentiation and, at the same time, precluding background reactivity that produces racemic products would be difficult, and (2) substitution reactions catalyzed by Brønsted bases are usually sluggish due to poor turnover; leaving groups are poorly basic and often cannot deprotonate the nucleophile or the protonated base.^20,21^ To put it in another way, the less acidic the substrate, the slower the catalyst turnover in Brønsted base catalysis.

We developed two different S_N_Ar methods with a distinct catalyst turnover (Scheme 1). The first one started with N–Si nucleophiles (Si-Nu-3), inspired by the work of Thiel and coworkers,^22^ and the second, directly from N–H heterocycles (H-Nu-2) and promoted by BEE-2, namely trifluoromethyltrimethylsilane^23^ (Ruppert–Prakash reagent). The fluoride catalyst is released in solution by air- and moisture-stable commercial tetrabutylammonium difluorotriphenylsilicate (TBAT). As it turns out, the synthesis of 3 was complete in 30 s by either method employing E-2.

**Scheme 1:**
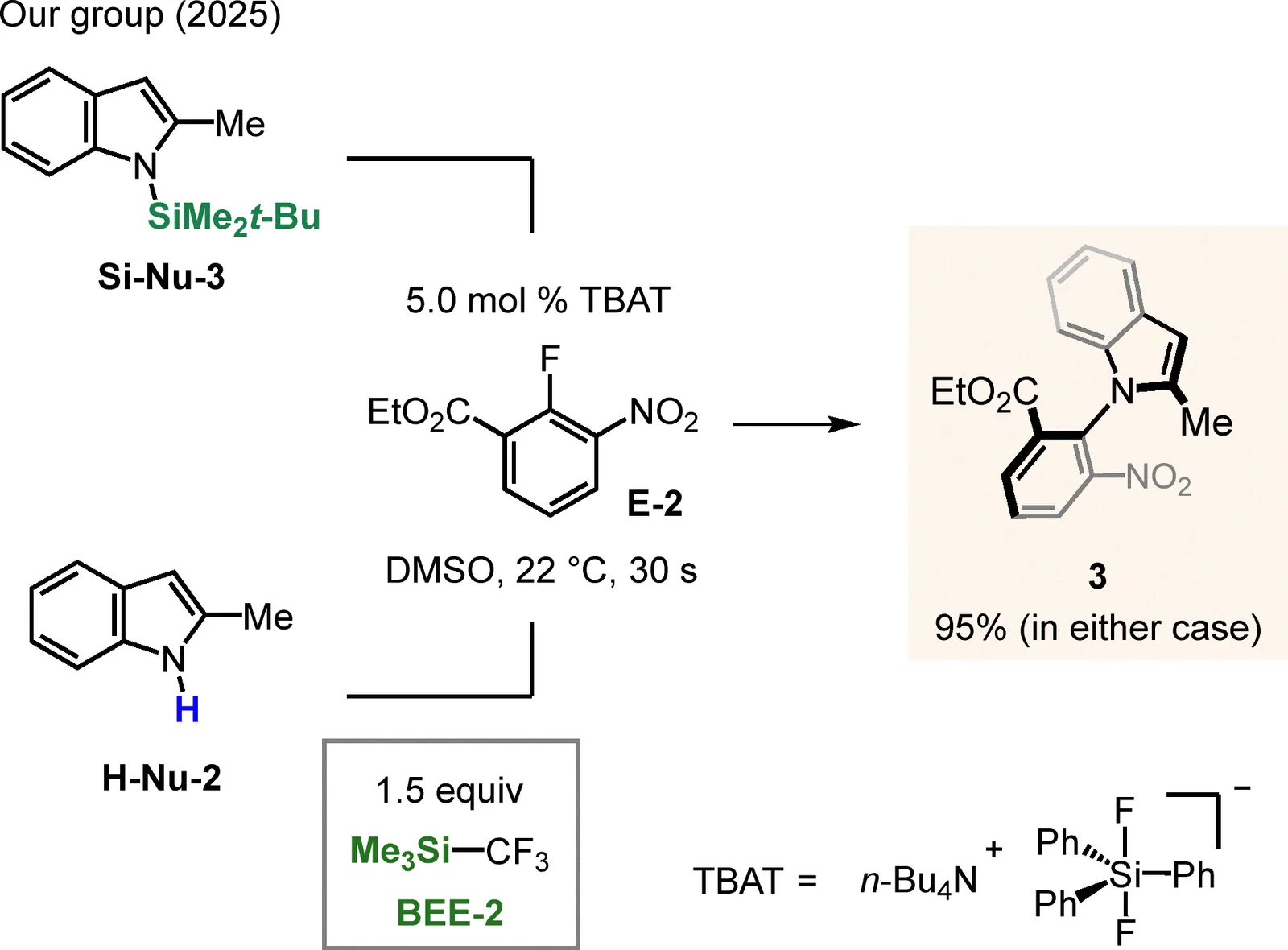
Fluoride-catalyzed nucleophilic aromatic substitution reactions with N–Si and N–H heterocycles enable efficient synthesis of N–C atropisomers.

The review has been divided into two main sections. In the first one, the silane acts as a pro-nucleophile in catalytic nucleophilic substitution (Fig. 2(A)) or addition (Fig. 2(B)) reactions. In the second, the silane is a base-embedding electrophile that drives catalytic deprotonation, once again for substitutions (Fig. 2(C)) or additions (Fig. 2(D)). The abovementioned S_N_Ar reactions reported by us are instances of substitution reactions. For substitution, in general, an anionic Lewis base catalyst (X_cat_^−^Org^+^) reacts with an organosilane (Nu–Si) to give the reactive intermediate (Nu^−^Org^+^), which then undergoes substitution with the electrophile (X_cat_–E), releasing the desired product (Nu–E) and regenerating the catalyst (Fig. 2(A)). Alternatively, the catalyst (X_cat_^−^Org^+^) reacts with a silicon-containing base-embedded electrophile (Si–B), leading to generation of the base *in situ* (B^−^Org^+^) and then the reactive nucleophile (Nu^−^Org^+^) *via* deprotonation (Fig. 2(C)). The formation of the product releases the catalyst (X_cat_^−^Org^+^), which is ready for turnover by reacting with the BEE. The addition processes are represented by the aldol reactions developed by Heathcock, Noyori and Kuwajima (Fig. 1). The main distinction is that, in addition reactions, the Nu^−^Org^+^ intermediate reacts, incorporating the electrophile to form Nu–E^−^Org^+^, which then turns over the catalytic cycle by reaction with the organosilane pro-nucleophile (Fig. 2(B)) or the base-embedding electrophile (Fig. 2(D)). The product of addition contains the silyl group (Nu–E–Si), which often is cleaved *in situ* or during workup.

**Fig. 2:**
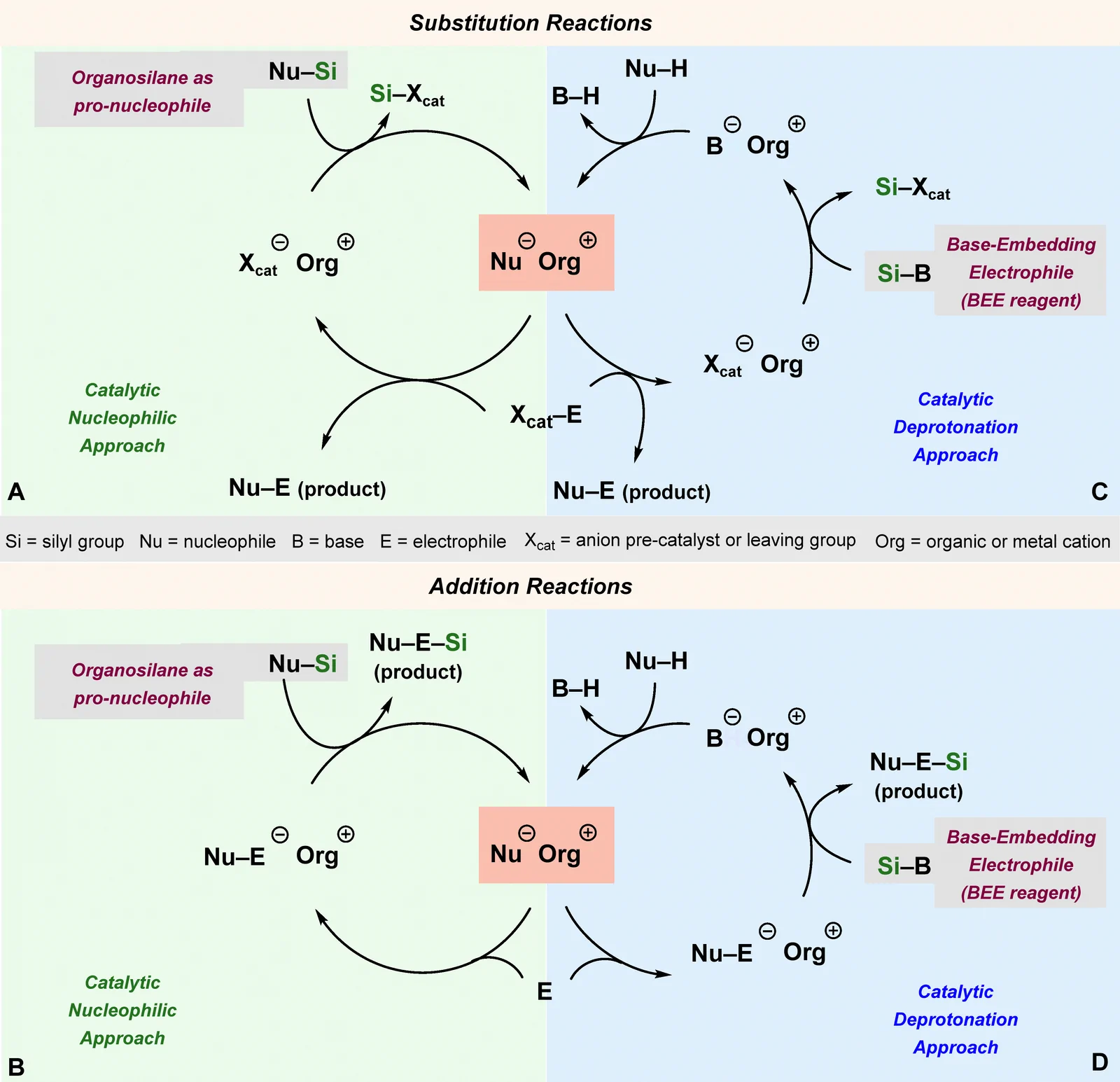
Anions as catalysts. General catalytic cycles for: (A) substitution reactions employing organosilanes as pro-nucleophiles. (B) Addition reactions employing organosilanes as pro-nucleophiles. (C) Substitution reactions employing organosilanes as pro-bases. (D) Addition reactions employing organosilanes as pro-bases.

One important mechanistic consequence, applicable to both substitution and addition processes, is that the anion added to the reaction is formally a pre-catalyst, which, by reacting with the organosilane, can lead to the formation of the desired organic product. The catalytically active species in substitutions is the leaving group, for example fluoride in S_N_Ar. For additions, it is the anion generated upon reaction between the anionic nucleophile and the electrophile, for instance, the alkoxide intermediate in aldol reactions. In Fig. 2B and D, the activation of the pre-catalyst has been omitted but it takes place as in Fig. 2A and C, giving rise to Nu^−^Org^+^. We also note that the catalytic cycles in Fig. 2 display reactive intermediates in general terms as, apart from highlighting catalyst turnover, our main goal is to highlight synthesis possibilities in connection with potential future developments.

## 2. Organosilanes as pro-nucleophiles

The Tan group developed bisguanidinium-based chiral catalysts to promote enantioselective reactions through formation of enantioenriched ion pairs.^24,25^ In one such reaction, silyl ketones (Si-Nu-4) were activated by bisguanidinium fluoride (F^−^bgn-1^+^, prepared *in situ* from the corresponding chloride) and underwent enantioselective 1,2-anionotropic rearrangement, followed by stereospecific Brook rearrangement assisted by the silanol, for synthesis of secondary silyl ethers and alcohols (4) after desilylation (Scheme 2).^26^ Note that 4.0 equiv. of CsF was employed, thus promoting efficient salt metathesis between bgn-1 and CsF and controlling undesired background reaction catalyzed by a Cs^+^-containing nucleophile were key to high enantioselectivity. It merits mentioning that Brook rearrangement, followed by elimination and intermolecular aldol, was employed in another context by Campagne,^27^ Leclerc^28^ and their coworkers.

**Scheme 2:**
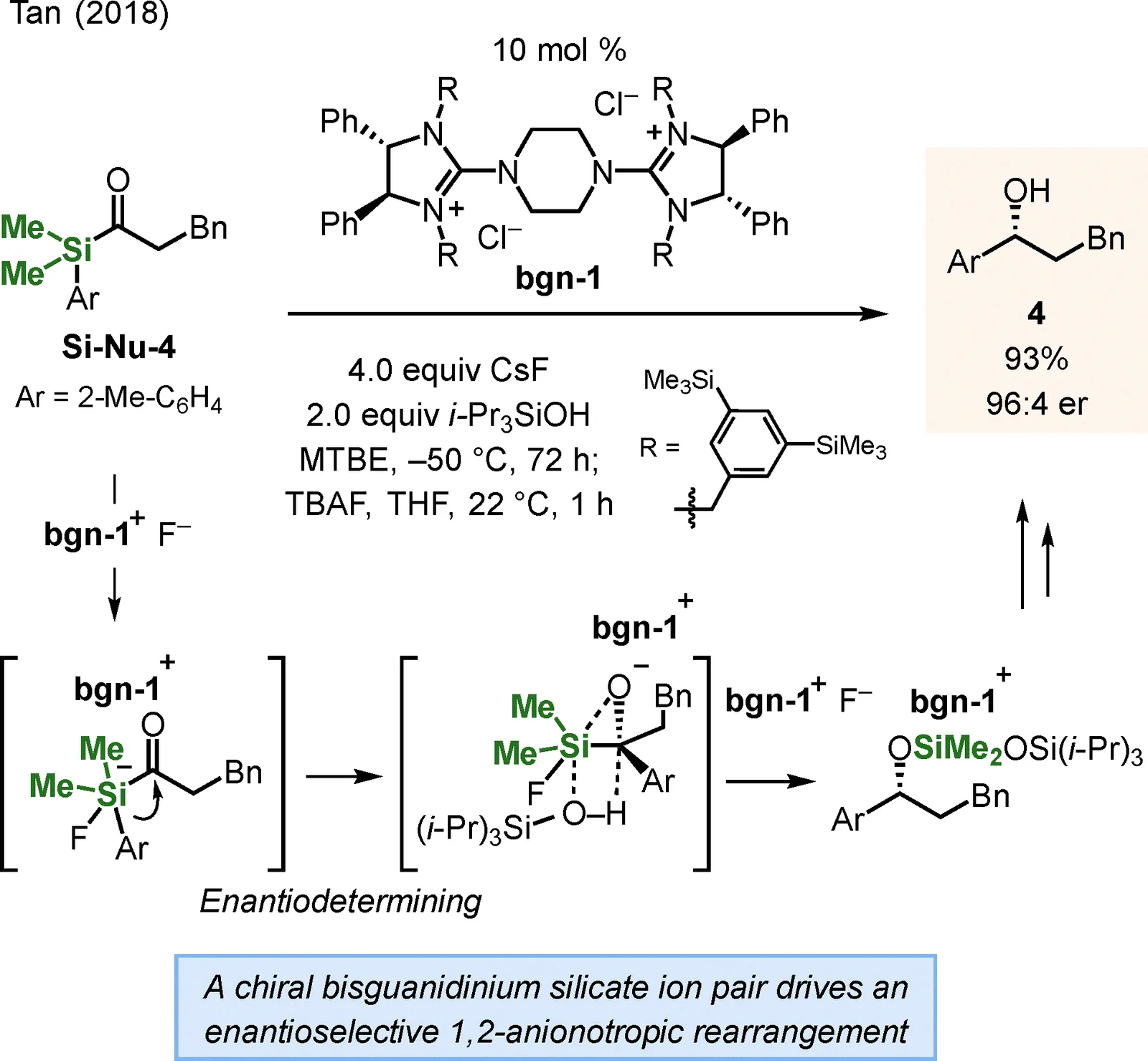
Salt metathesis can generate a chiral fluoride ion pair and promote enantioselective rearrangements of fluorosilicates.

Racemic allylic fluorides, derived from the Morita–Baylis–Hillman (MBH) reaction,^29^ have been employed as electrophiles in enantioconvergent substitution reactions to forge C–N, C–C and C–O bonds (Scheme 3). The stereoablation is caused by the S_N_2′ reaction of the tertiary amine portion of the catalyst ((DHQD)_2_PHAL) with the allylic fluoride (E-3), giving rise to an enantioenriched ammonium fluoride ion pair (*in situ*-cinc-F), as shown by Shibata early on.^30^ In connection with the concept of organosilanes as latent pro-nucleophiles,^14^ Vilotijevic reported that *N*-silyl indole derivatives (Si-Nu-5) undergo another allylic substitution with the ion pair, forming 5 and regenerating the neutral catalyst.^31^ Shibata,^32^ Vilotijevic^33^ and Companyó^34^ groups leveraged a similar strategy instead, to forge C–C bonds: the silyl-substituted difluoroester Si-Nu-6 and the cyclic silyl enol ethers Si-Nu-7a-b react to furnish 6 and 7a-b, respectively. Vilotijevic also showed that the strategy using Si-Nu-7a can lead to kinetic resolution of MBH allylic fluorides (recovered with 99 : 1 er).^33^ Carrying out enantioselective MBH,^35^ as well as its aza-version^36^ is quite challenging. The Vilotijevic group promoted enantioconvergent reactions with a variety of silyl carbamates (Si-Nu-8)^37^ and silyl ethers (Si-Nu-9),^38^ respectively, toward synthesis of compounds such as 8 and 9, which could in principle be deprotected. The overall strategy is therefore an alternative to direct enantioselective MBH reactions. One advantage of the reactions in Scheme 3 is that enantioselectivities were high and somewhat independent of the nucleophile.

**Scheme 3:**
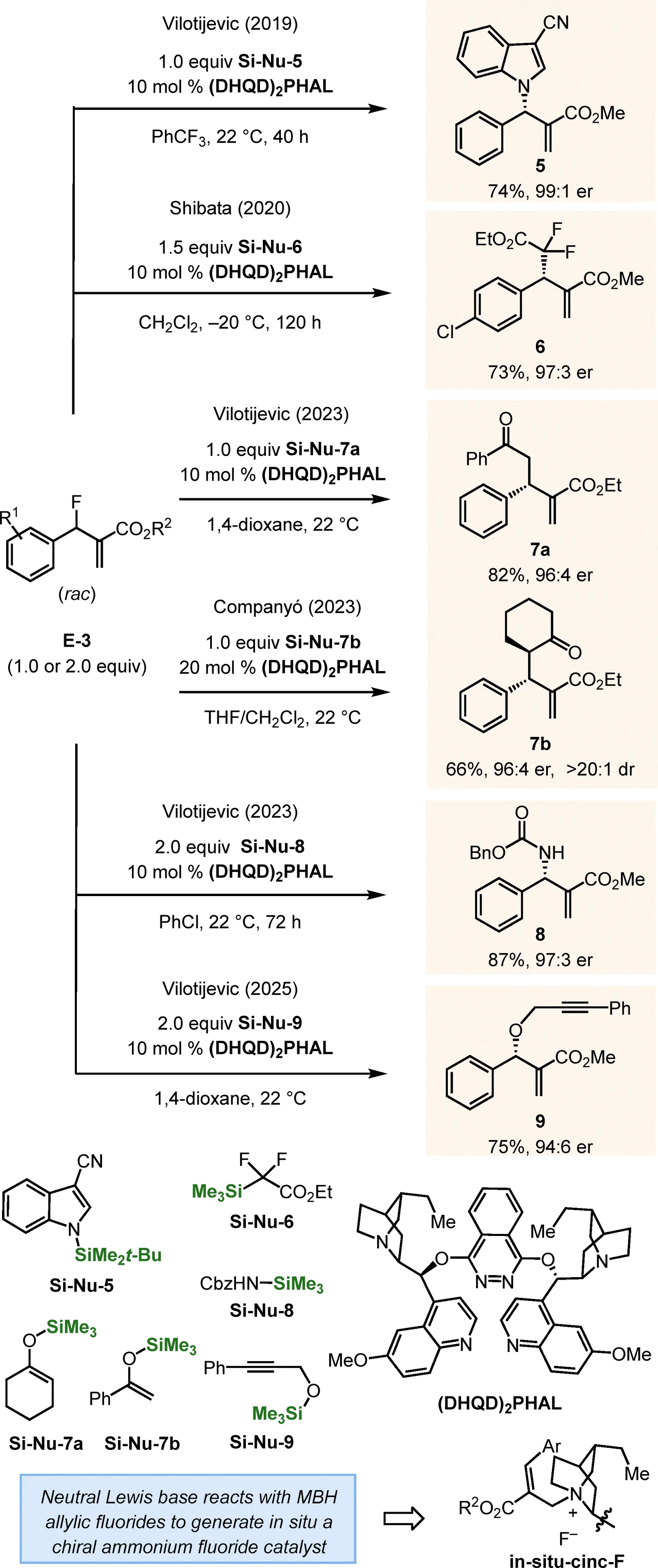
Enantioconvergent C–C, C–N and C–O bond formation by substitution on Morita–Baylis–Hillman allylic fluorides.

Bandar and coworkers reported substitutions at C–F in trifluoromethyl-substituted arenes (E-4 and E-5, Scheme 4). In the initial discovery, trimethylallyl silane (Si-Nu-10) was employed as the nucleophile for synthesis of 10.^39^ The reaction is catalyzed by CsF in the presence of a crown ether (90% yield) but only poorly by the P_4_-*t*-Bu base (16% yield). S_N_2 reactions at CF_3_-groups are unlikely. The authors therefore provided evidence for a radical mechanism, involving single electron transfer from the fluoroallyl silicate intermediate to the trifluoromethyl arene, causing a heterolytic C–F cleavage and formation of a difluoroaryl methane radical, which is trapped by the allyl silicate. Later, the same group employed a supersilane, tris(trimethylsilyl)silane (Si-Nu-11), to promote other C–C formations, including with 4-formylmorpholine (4-FM).^40^ The supersilane promoter leads to formation of 11*via* the organosilane Si-Nu-12. Firstly, formate, a pre-catalyst, promotes the silylation toward Si-Nu-12 and then an alkoxide, formed on the C–C formation event, catalyzes the formation of 11. A crown ether is necessary, likely to increase the rate of single electron transfer through a looser ion pair.

**Scheme 4:**
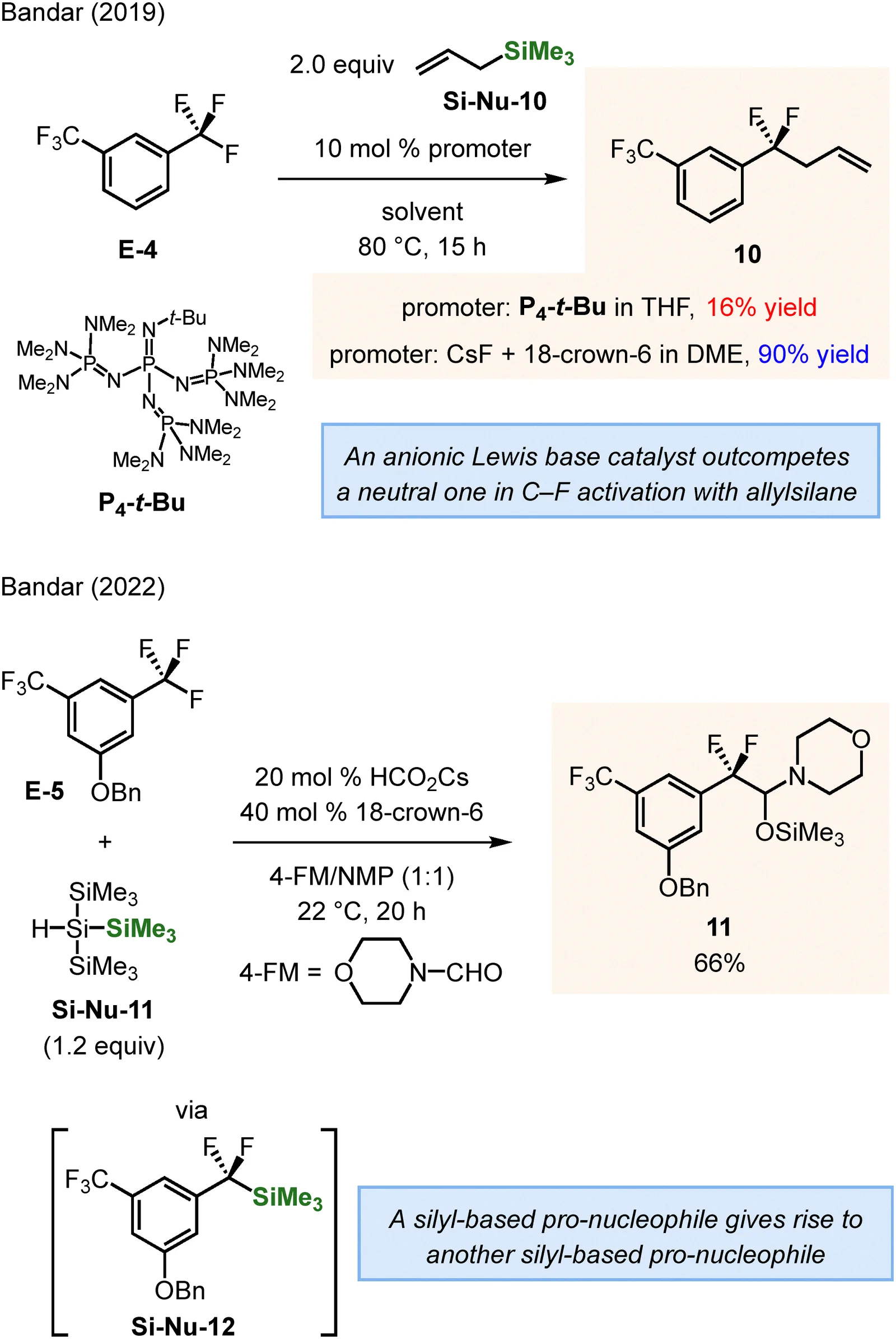
C–C formation by substitution on C–F of trifluoromethyl arenes.

Silyl-substituted azobenzenes are mild precursors for aryl and alkyl anions, as shown by Oestreich^41^ and Chauvier.^42^ Oestreich and coworkers employed the reactivity of Si-Nu-13 in two ways (Scheme 5). First, for addition to aldehydes, by a process initiated by silanolate and catalyzed by an alkoxide for synthesis of 12,^43^ and second in fluoride-catalyzed substitution of 2-trifluoromethyl styrenes for synthesis of 13.^44^ Upon catalytic anionic nucleophilic activation leading to the loss of nitrogen, silyl-substituted azobenzenes can give rise to reactive organometallic nucleophiles.

**Scheme 5:**
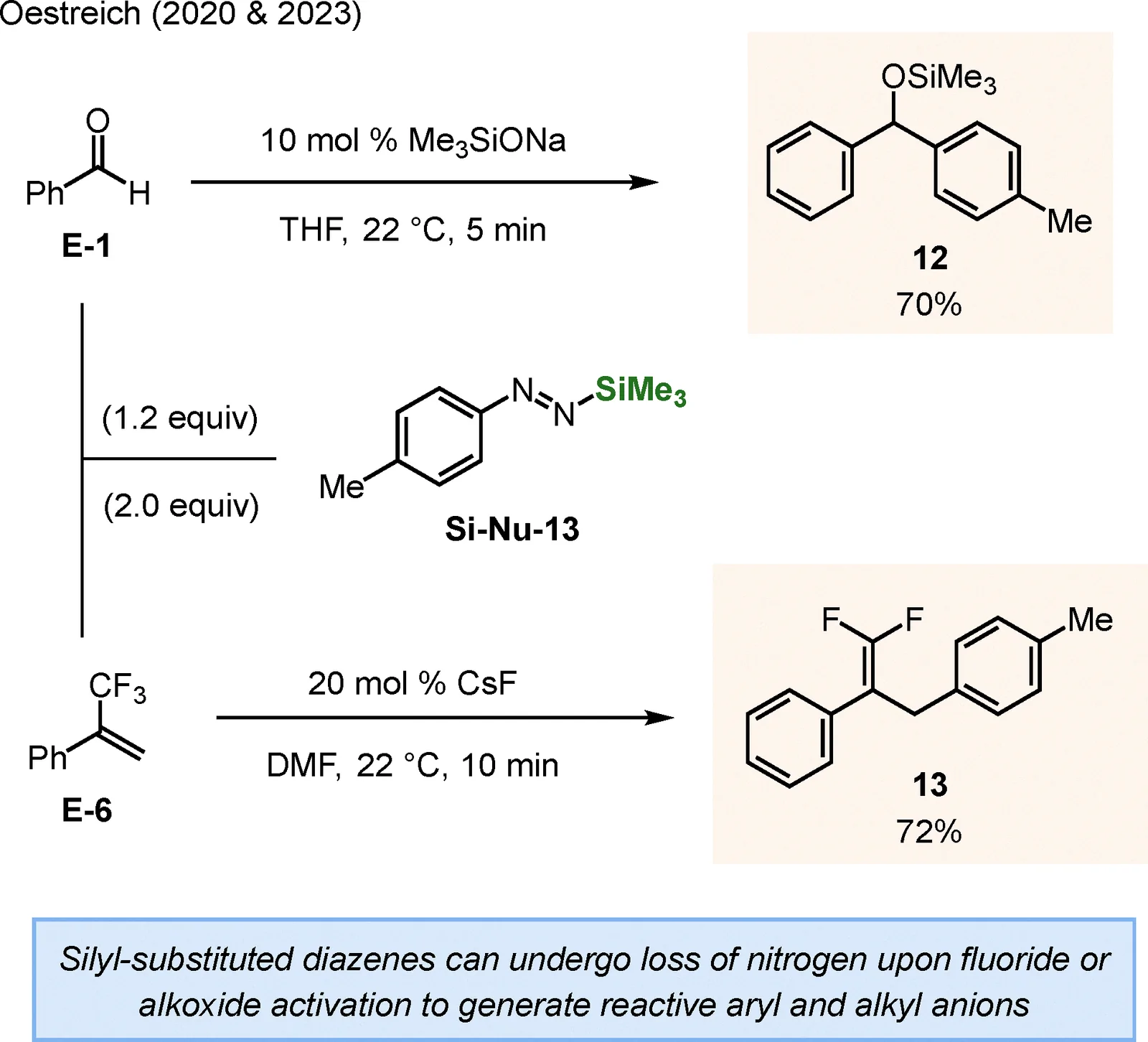
Catalytic aryl group addition and substitution with silicon-based azoarenes.

Deng and coworkers developed an enantioconvergent and diastereoselective conjugate addition strategy by reacting silyl substituted imines (Si-Nu-14) with cyclic and acyclic α,β-unsaturated ketones (E-7, Scheme 6).^45^ The catalytic reaction takes advantage of a chiral ammonium fluoride pre-catalyst,^46^ which can be prepared *in situ*, from an ammonium iodide *via* salt metathesis with Amberlyst A-26 fluoride resin, or preformed. In such conjugate addition, the enolate is likely responsible for catalyst turnover. The fluoride resin is needed in both cases, and the nature of the ammonium source changes the reaction outcome drastically. The ammonium fluoride, compared to the iodide, leads to a higher yield (86% *vs.* 40%), diastereoselectivity (88 : 12 *vs.* 85 : 15 dr) and a higher ratio of the desired to the undesired product (>95 : 5 *vs.* 70 : 30 15 : 14). The authors mentioned that the ammonium fluoride, which is synthesized by salt metathesis between AgF and the corresponding ammonium iodide in MeCN, should be stored at −40 °C. In ^19^F NMR, the catalyst solution in MeOD-*d*_3_ displays two peaks with chemical shifts at −133 ppm, corresponding to fluoride, and at −177 ppm to likely bifluoride or MeOD-stabilized fluoride species. The strategy of conjugate addition has also been disclosed by Denmark employing 2-silyl-1,3-dithianes as nucleophiles.^47^

**Scheme 6:**
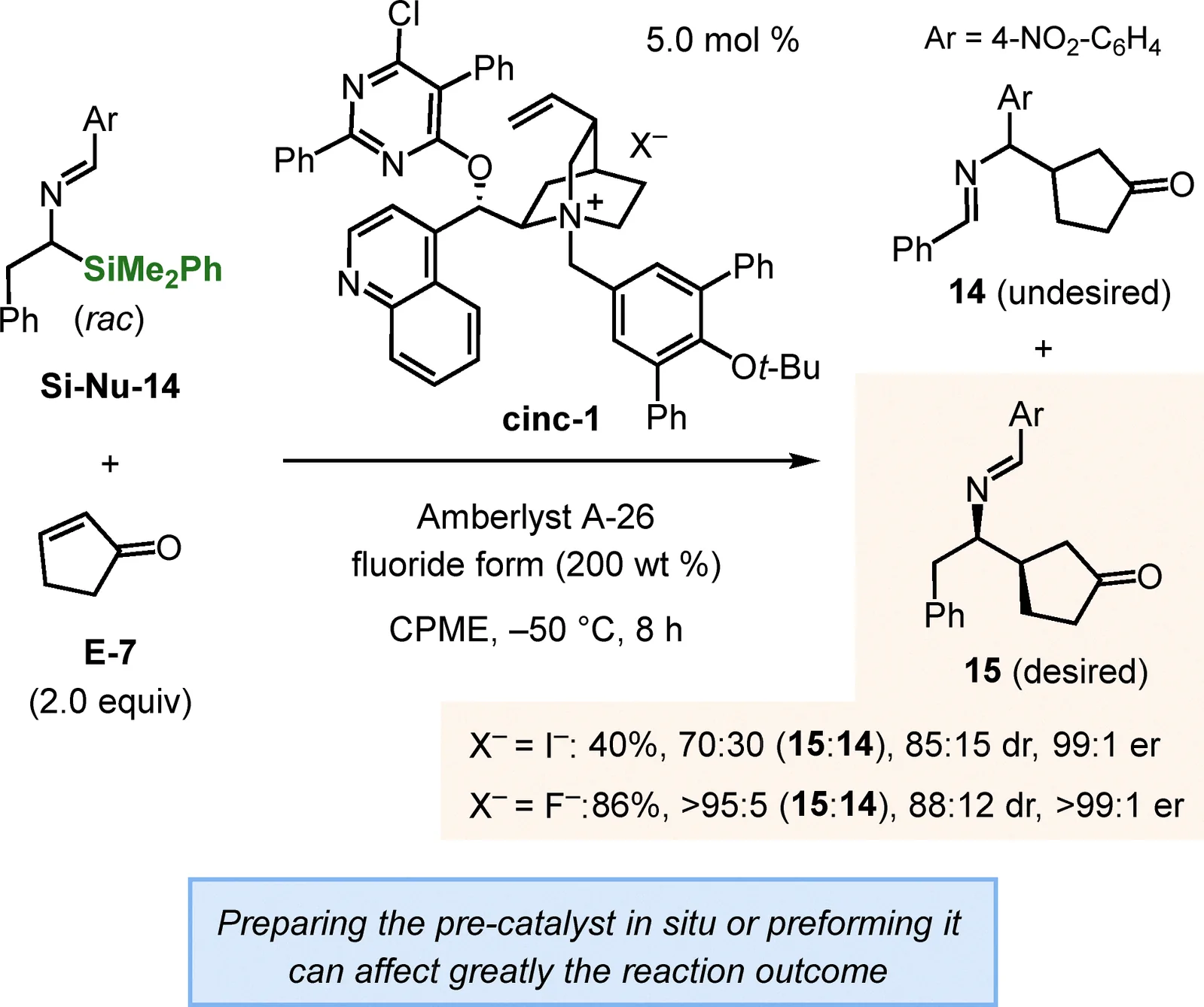
Catalytic enantioselective conjugate addition promoted by a chiral ammonium fluoride.

2-Trimethylsilylpyridine (Si-Nu-15) can react with aryloyl fluorides (E-8) to form α-silyl acyl pyridinium fluoride salts, as shown by Barbasiewicz's group (Scheme 7).^48^ Through the action of a fluoride catalyst, the pyridinium fluoride can undergo silyl group loss to generate a zwitterion that is prone to 1,2-acyl shift toward ketones (16). Similarly, Le and coworkers reported that carbamoyl fluorides can react with alkynyl silanes to forge propionyl amides *via* catalysis with fluoride.^49^

**Scheme 7:**
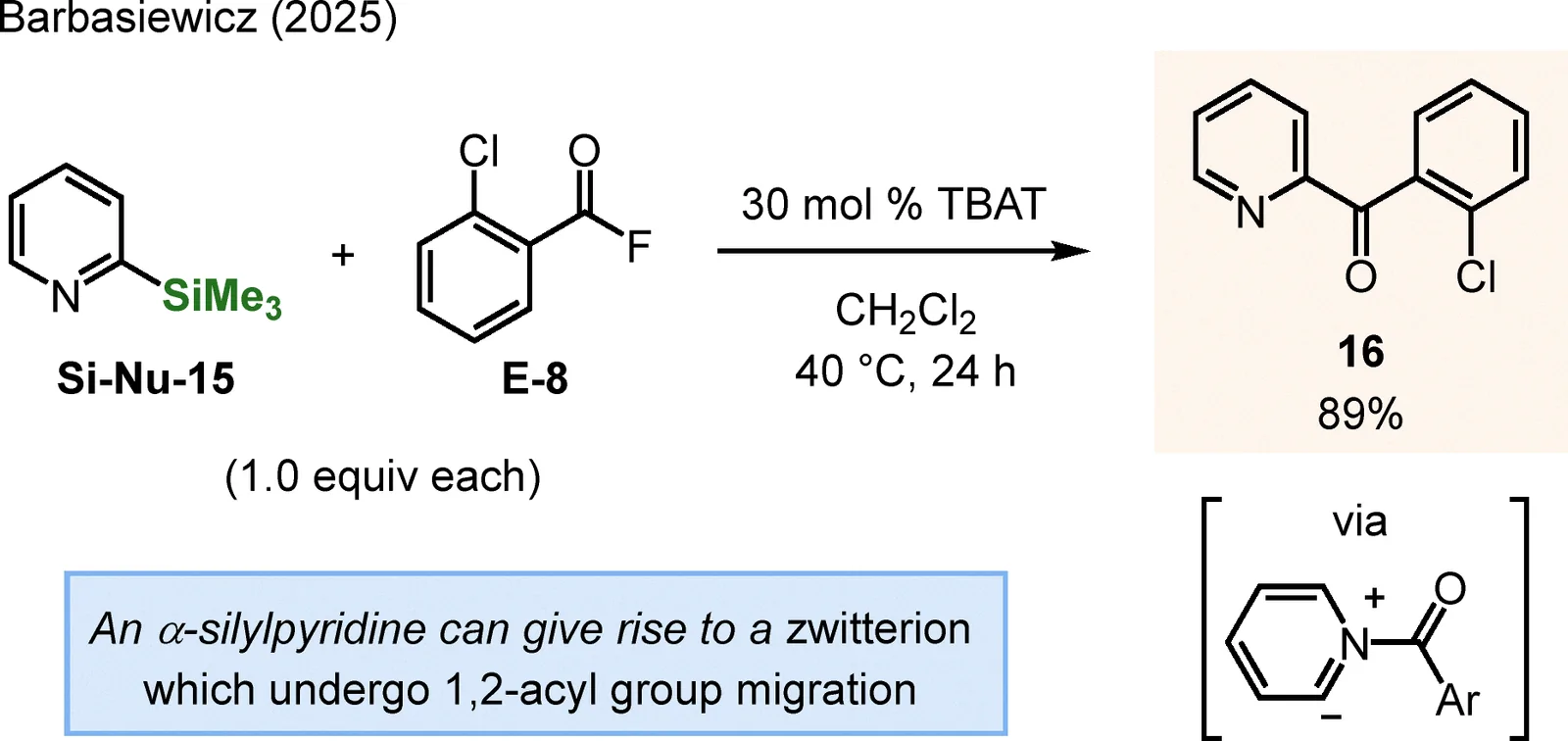
Fluoride-catalyzed 1,2-acyl group migration of α-silyl acyl pyridinium salts.

## 3. Organosilanes as base-embedding electrophiles

Shibata and coworkers disclosed a unique intramolecular substitution having a fluoride leaving group and a carbamate nucleophile (Scheme 8).^50^ Stoichiometric amounts of strong inorganic and organic bases, such as NaH and phosphazene P1-*t*-Bu, promoted cyclization of H-Nu-3 for the synthesis of the 2-oxazolidinone *rac*-17 at 22 °C in THF, in moderate yields (56–59%) in 17–24 h. By employing 10 mol% of CsF (or Cs_2_CO_3_) at 22 °C in MeCN, in the presence of *N*,*O*-bis(trimethylsilyl)acetamide (BSA, BEE-3), the yield improved to 94%, and the reaction was complete in 1 h. These results suggest that the use of an anionic Lewis base catalyst in combination with a BEE is not simply a replacement for strong basic conditions. Stoichiometric traditional Brønsted base catalysis, especially that carried out at room temperature, can often lead to byproducts. It is not straightforward to explain why the catalytic reaction is superior; however, it is possible that the cation plays a role, with Cs^+^ being the optimal, or that the silicon atom activates the C–F cleavage.

**Scheme 8:**
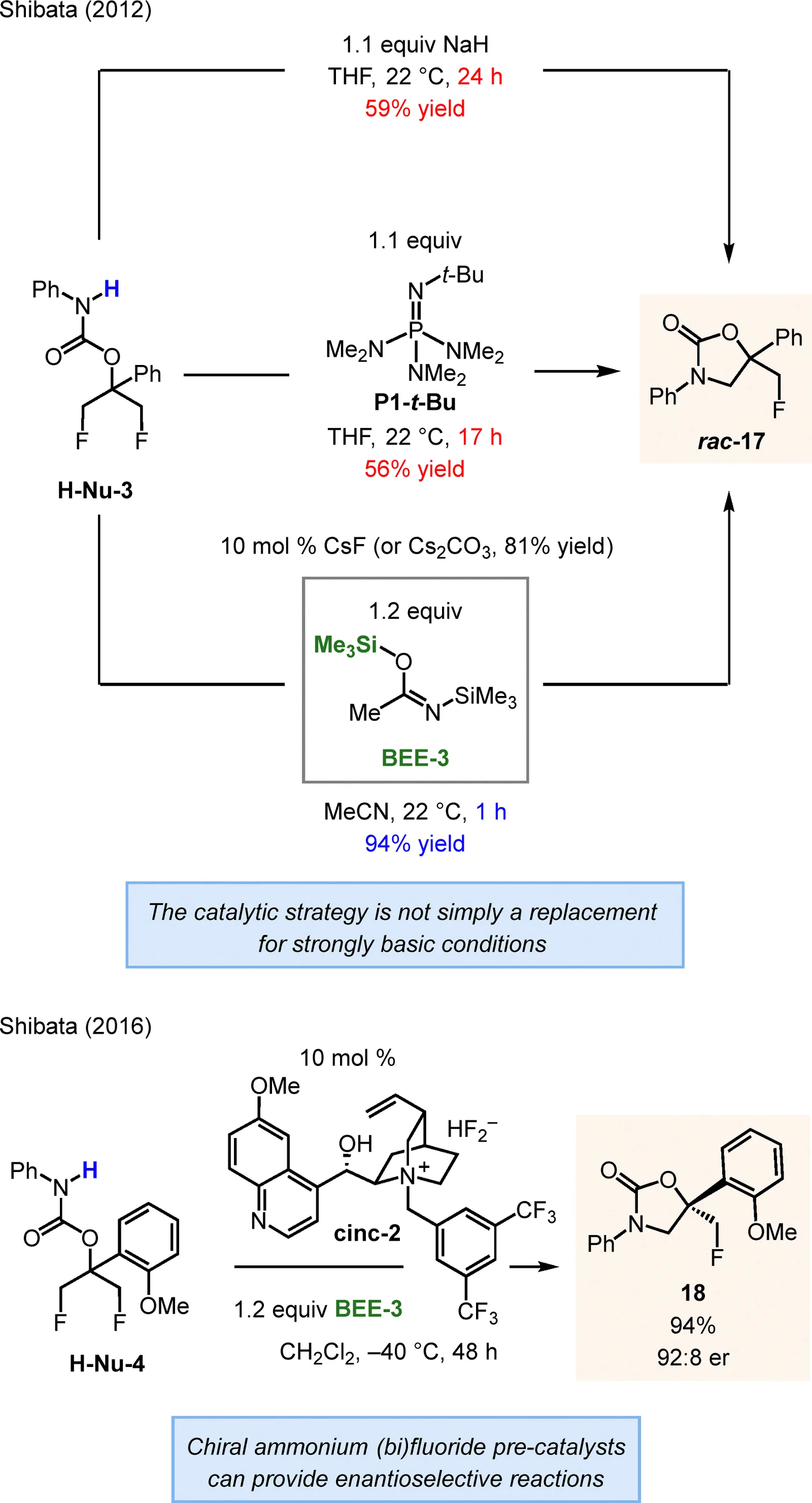
Catalytic intramolecular substitutions with a fluoride acting as the leaving group and the corresponding enantioselective reaction with a chiral bifluoride pre-catalyst.

Four years after their initial report, the Shibata group released the catalytic enantioselective variant of their substitution (Scheme 8).^51^ It turns out that catalytic amounts of cinc-2, a chiral bifluoride, can serve as a pre-catalyst. Cryogenic temperature (−40 °C) and longer reaction times (48 h) were employed to improve selectivity and yield. The authors optimized the reaction by preparing the pre-catalyst *in situ*, through salt metathesis between readily available ammonium bromides and AgF. In general, an ammonium bifluoride is more stable and easier to handle than the corresponding fluoride, making the use of bifluorides superior in cases where they initiate catalysis. Perhaps that was not the case in the abovementioned reaction by Deng (Scheme 6).^45^ The Shibata group also showed that epoxides can be formed from 2,2-difluoroalcohols by substitution.^52^

Levacher and colleagues developed catalytic enantio- and diastereoselective Henry reactions; for example, for synthesis of 19 starting with nitroalkane H-Nu-5 and aliphatic and aromatic aldehydes such as E-9 (Scheme 9).^53^ Apart from BEE-3, the authors employ the ammonium chloride cinc-3, which alone is unable to turnover the catalytic cycle but undergoes salt metathesis with sodium 4-methoxyphenoxide to generate the active pre-catalyst, a chiral ammonium aryloxide. In this scenario, controlling background is challenging but was made possible, at least partly, as good enantioselectivities were achieved for the given example (83 : 17 er). The authors pointed out that the aryloxide, as well as the resulting alkoxide formed upon addition, can act as a Brønsted base. This is plausible because the reactions work to some extent without BEE-3 (35–45% yield). However, higher conversions are obtained in the presence of BEE-3, suggesting that both the aryloxide and alkoxide can more rapidly turnover the catalytic cycle by reacting with the silane. In the earlier work by the Levacher group on the catalytic enantioselective vinylogous aldol reaction,^54^ which was also initiated by chiral ammonium 4-methoxyphenoxide as a pre-catalyst, the base-embedding electrophile reagent was essential. Cavallo and Della Sala developed a conjugate addition strategy in which hydroxide, from KOH/18-crown-6, is the catalyst precursor.^55^

**Scheme 9:**
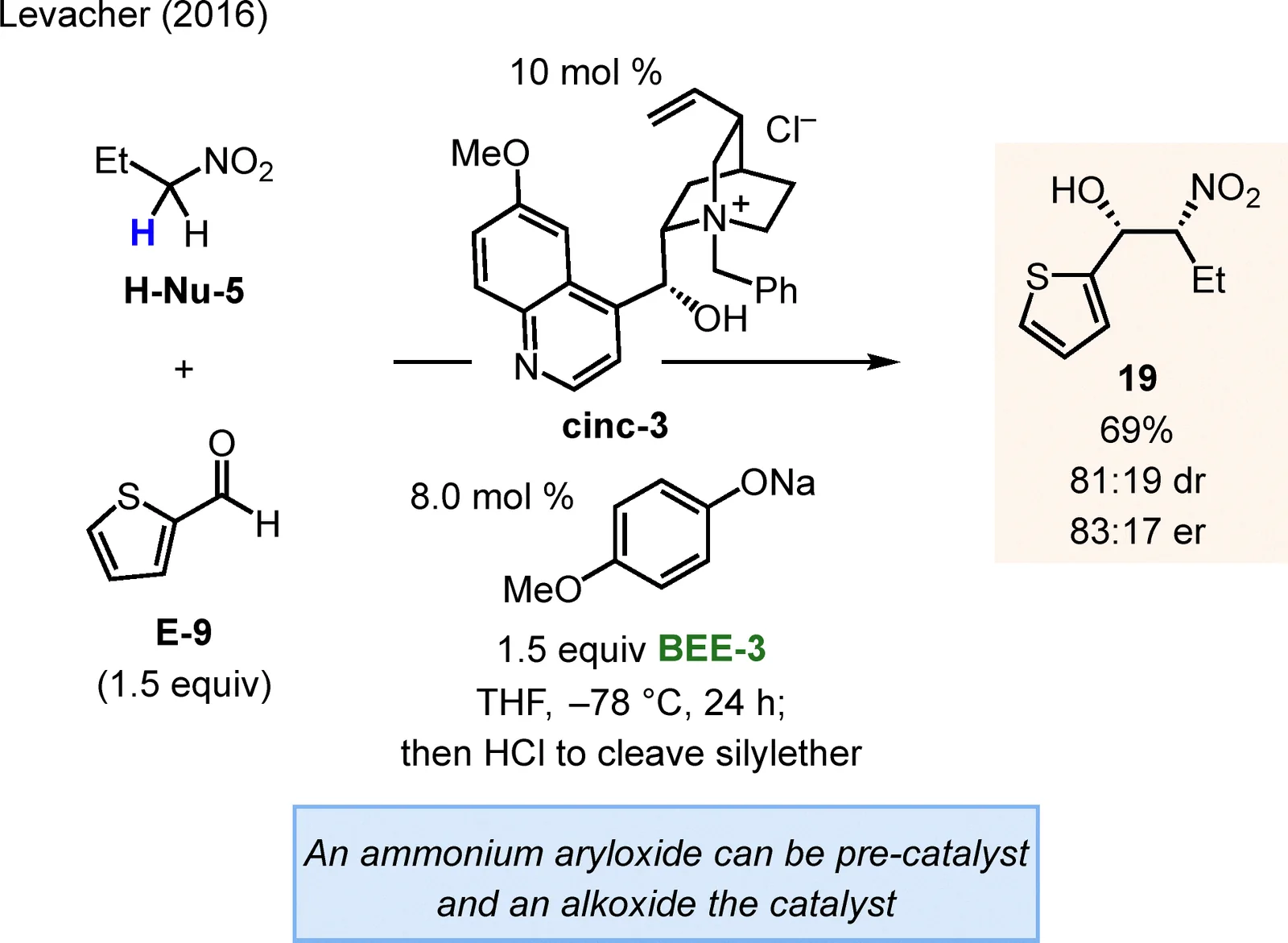
Catalytic enantioselective Henry reaction with an aryloxide pre-catalyst.

Apart from bisguanidinium catalysts (Scheme 2), the Tan group developed pentanidium-based ones.^56^ They employed 5.0 mol% of pentanidium chloride ptn-1 and 2.0 equiv. CsF to generate *in situ* a chiral fluoride catalyst responsible for promoting enantioselective benzylation of racemic H-Nu-6 (Scheme 10). Under optimal conditions, employing BEE-3 in THF as the solvent and at −40 °C, background reactivity was not operative or negligible as synthesis of 20 was accomplished with 98 : 2 er. Distinct base-embedding electrophiles were shown to impact reactivity and selectivity. With tris(trimethylsilyl)amine (BEE-4), 10 mol% ptn-1 and at −20 °C, the selectivity toward 20 is diminished (90 : 10 er). Control experiments with catalytic tetrabutylammonium iodide in combination with inorganic bases, *en route* to *rac*-20, leads to sluggish or side reactions such as lactone opening. This is another testament to the crucial role of the fluoride-catalyzed process. In the same work, the authors displayed a series of other fluoride-catalyzed enantioselective reactions. One was for the synthesis of 21 from H-Nu-7 and E-11, having bisguanidinium chloride bgn-2 as a catalyst precursor and a slightly modified version of BEE-3, namely BEE-5. Other reactions, employing allylic electrophiles as well as employing acyclic silyl enol ethers have been reported by Tan.^56^ Allylic transposition *via* rearrangement has been disclosed earlier by Craig and coworkers, and proposed to proceed through analogous mechanisms.^57^

**Scheme 10:**
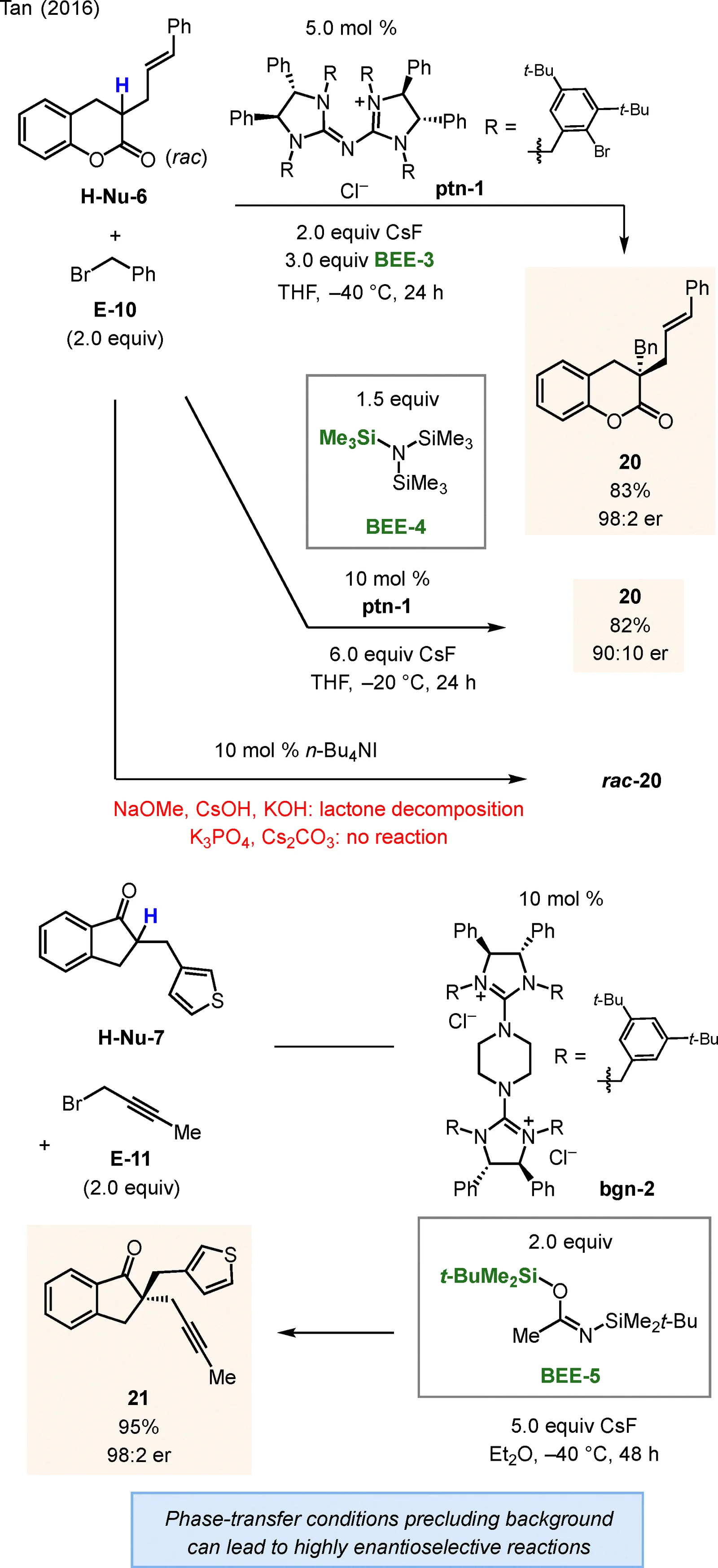
Catalytic enantioconvergent alkylations under phase-transfer conditions with pentanidium and bisguanidinium catalysts.

Early on, Kondo and Inamoto and colleagues investigated reactions promoted by substoiochiometric amounts of fluoride sources and BEE reagents.^58,59^ Later, the Kondo and Shigeno group showed several catalytic deprotonations of particularly weak acids.^60,61^ Direct α-deprotonation of H-Nu-8, promoted by catalytic tetramethylammonium fluoride and 2.0 equiv. BEE-4, followed by reaction with dimethylformamide (E-12), leads to substitution product 22 (Scheme 11).^62^ Benzylic deprotonation of H-Nu-9,^63^ as well as allylic deprotonation of H-Nu-10,^64^ lead to elimination products 23 and 24, respectively. E-12 can react in two different ways. If, after addition, the resulting alkoxide does not bear an α-proton, formylation by release of dimethylamine follows. If an α-proton is available, elimination takes place toward 23. A similar mechanism can be proposed toward 24 (in box), implying that a silanolate turns over catalysis.^65^ It is understandable that such deprotonations are carried out with BEE-4, giving rise to tetramethylammonium or cesium-based hexamethyldisilazide (Cs-HMDS), which, by forming a looser ion pair, are likely more reactive than Na- or K-HMDS.^66^

**Scheme 11:**
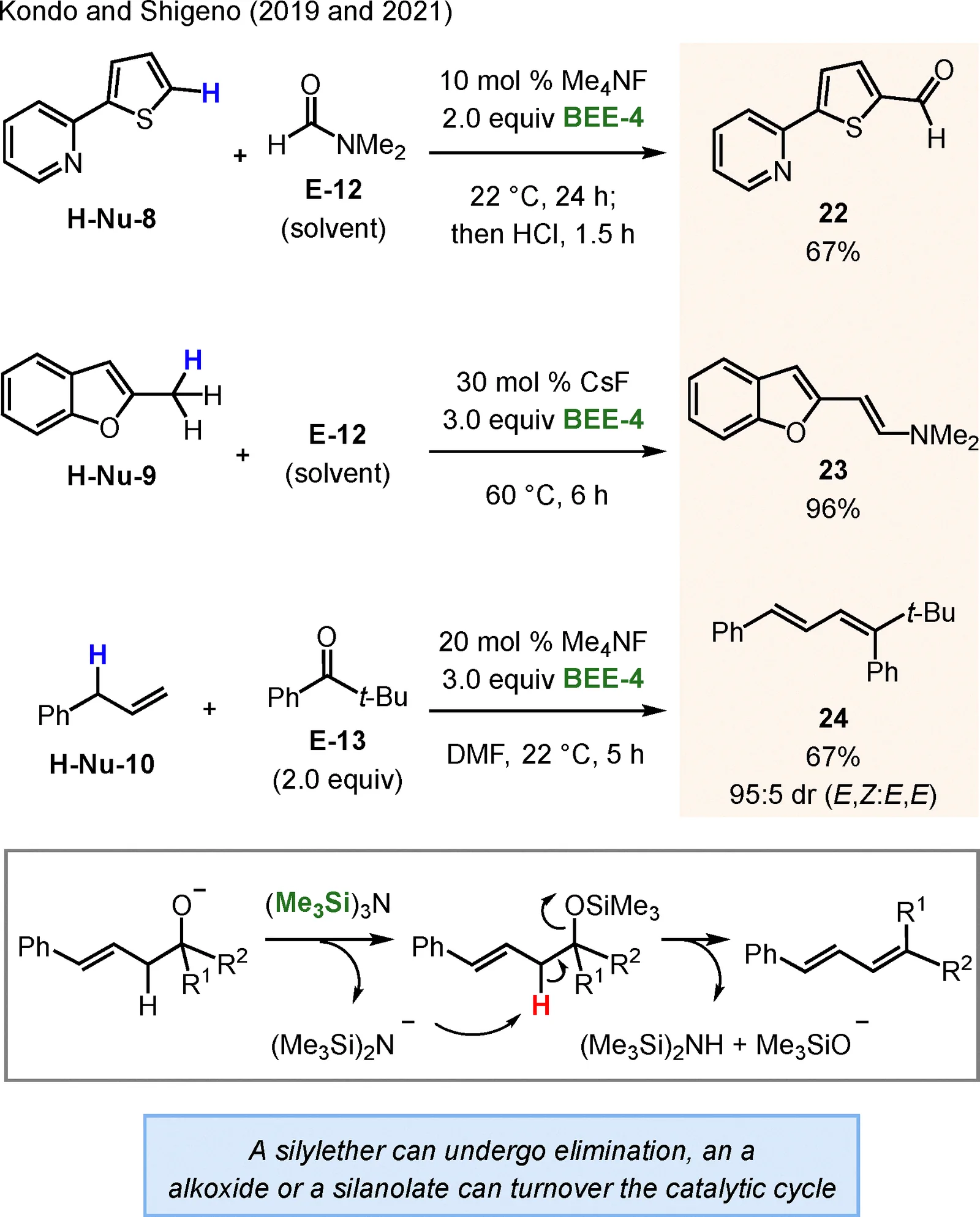
Catalytic deprotonation of aryl and benzyl C–H bonds.

Pihko and coworkers developed catalytic diastereoselective direct additions of diazoacetates (H-Nu-11) to aldehydes; one example being for the synthesis of 25 from E-14 (Scheme 12). Tetramethylammonium pivalate likely serves as a pre-catalyst, and the aldol reaction gives rise to an alkoxide-based catalyst.^67^ The Pihko group reported catalytic, carboxylate-based silylation of alkynes^68^ which, perhaps if combined with the alkynylation strategy reported by Le and coworkers,^49^ could lead to direct catalytic alkynylation reactions starting with terminal alkynes.^69^

**Scheme 12:**
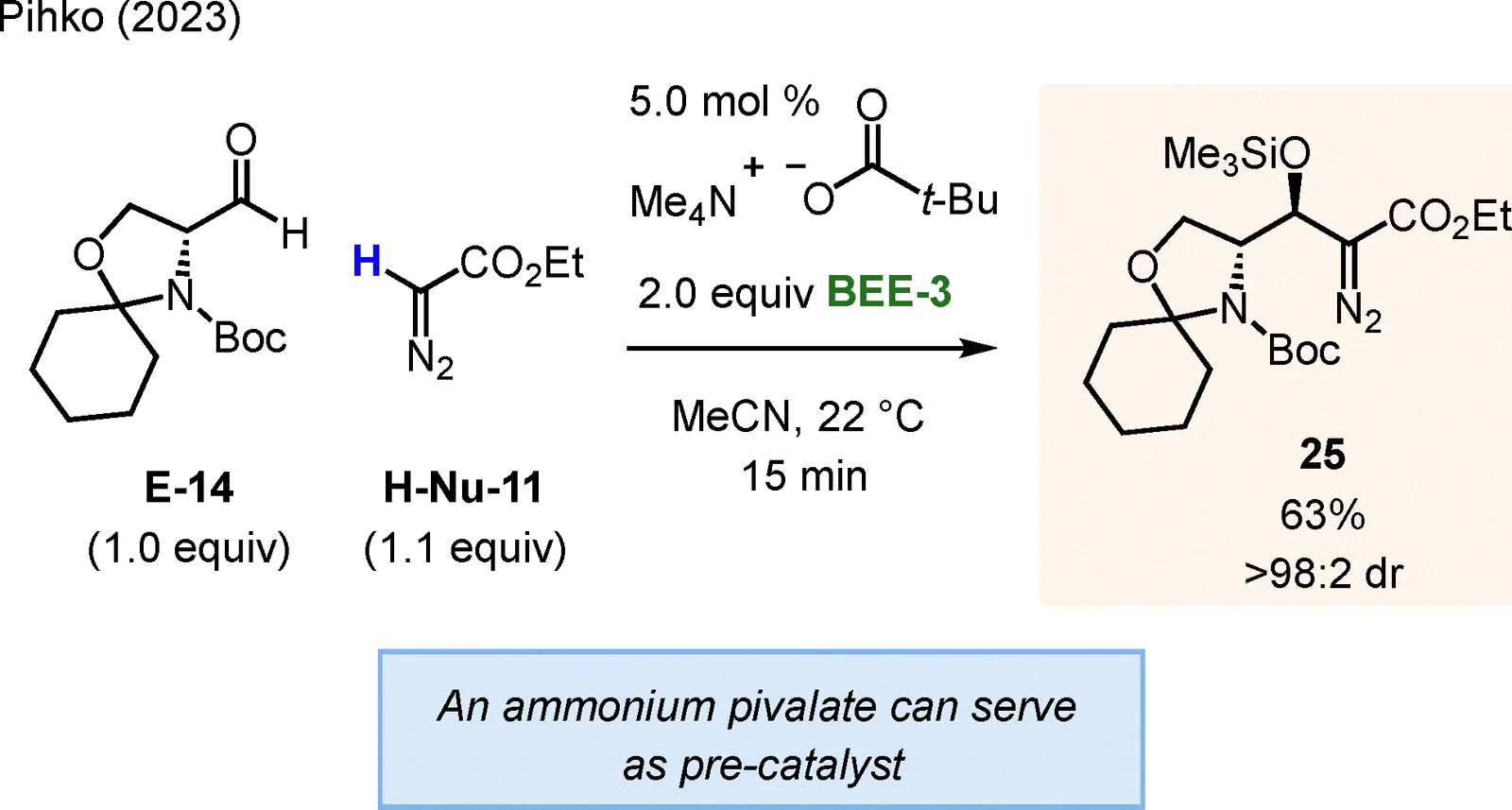
Catalytic deprotonation of α-diazoesters followed by aldol reaction.

As preliminarily discussed in the Introduction, we developed three methods for synthesis of heterobiaryl atropisomers based on the N–C stereogenic axis (Scheme 1)^19^ in which many heterocycles and fluoroarenes were employed (Scheme 13). Methods A and C employ N–H heterocycles (H-Nu-12); the difference is that the former is promoted by 2.0 equiv. Cs_2_CO_3_ and the latter by 5.0 mol% TBAT and 1.5 equiv. BEE-2. Method B employs N–Si heterocycles (Si-Nu-16) and catalytic TBAT.

**Scheme 13:**
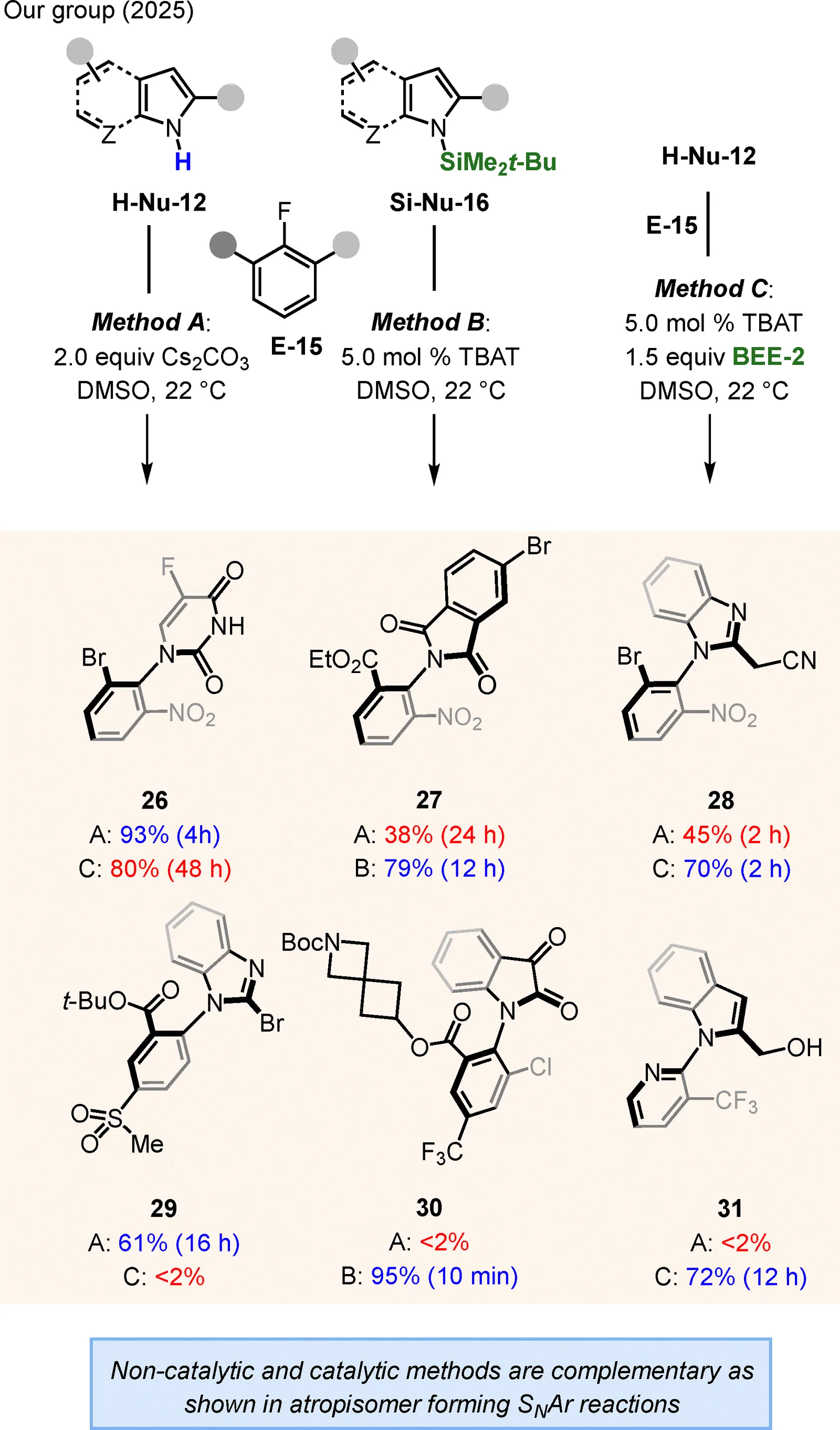
Synthesis of diversifiable N–C atropisomers by different S_N_Ar methods.

The methods are complementary, as suggested by the comparisons of the synthesis of atropisomers 26–31 (Scheme 13). N–Si heterocycles can be unstable, but when they are isolable, reactions can be efficient, for example toward bromophthalimide- and isatin-containing atropisomers 27 and 30. However, in the vast majority of cases, preparation of an organosilane is not needed and/or is difficult. In those cases, for example, for synthesis of atropisomers 26, 28, 29 and 31, the methods A and C are superior. Currently in our group, we determine experimentally which method, A or C, is superior for a given substrate. Method C can lead to faster reactions and control formation of byproducts, as Cs_2_CO_3_ can lead to undesired α-arylation of nitriles (28) or S_N_Ar on a primary alcohol (31). None of these issues apply to method C, however, there are cases in which method A is superior, for example, for synthesis of atropisomers 26 and 29.

The efficiency of our S_N_Ar, combined with increasing interest in cyclic atropisomers in drug discovery, led us to evaluate the possibility of carrying out catalytic macrocyclizations. By employing H-Nu-E-1 as substrates, with 10 mol% TBAT and 1.5 equiv. BEE-3 in THF or DMSO, we synthesized not only macrocyclic but also more strained medium-sized atropisomeric rings (32–35, Scheme 14).^70^ Apart from broad scope with respect to the ring size, the method allows incorporation of aromatic and aliphatic linkers, aromatic and aliphatic N-heterocycles and several drug-like and modifiable functional groups (nitro, nitrile, pyridine, ester, and halogens). Most reactions are carried out at 22 °C and are complete within minutes to a few hours. The approach is also efficient for peptide macrocyclization, which takes place *via* direct *N*-arylation of a tryptophan moiety (H-Nu-E-2) and leads to two atropodiastereomeric peptides, 36 with *S*_ax_ and *diast*-36 with *R*_ax_ configurations with low diastereoselectivity (66 : 34 36 : *diast*-36). Such macrocyclic compounds are relevant in the context of atropopeptides,^71^ naturally occurring and bioactive ribosomally synthesized post-translationally modified peptides (RiPPs).^72^ The RiPPs, isolated from natural sources, have been synthesized using Larock cyclization methods;^73,74^ our complementary approach is to provide stereochemical and structural diversity. Control experiments show that catalysis plays a major role in enabling the cyclizations, especially of linear substrates that are not biased for cyclization (aliphatic chains) and toward medium-sized rings.

**Scheme 14:**
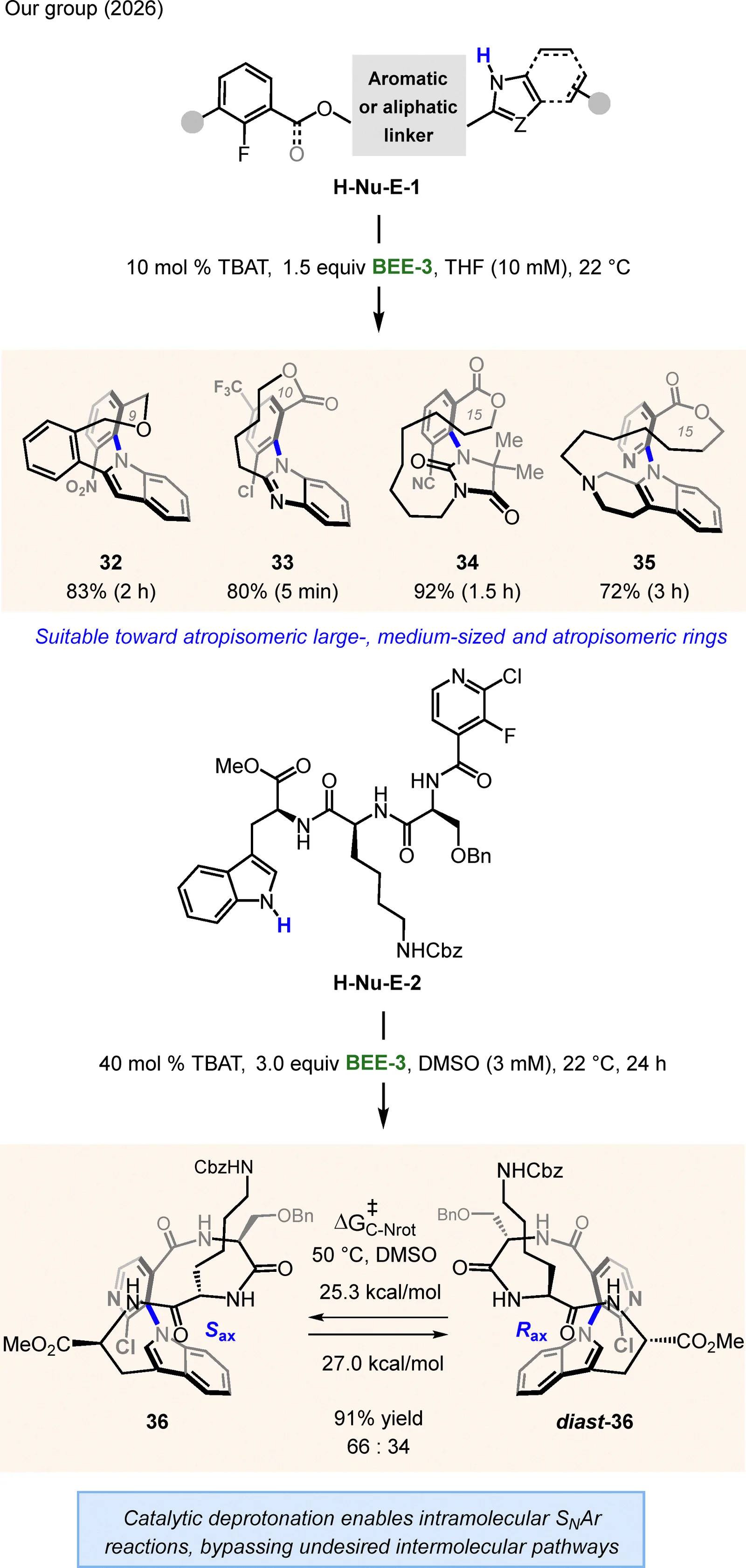
Synthesis of cyclic atropisomers by catalytic intramolecular S_N_Ar reactions.

## 4. Conclusions

The choice of whether to use an organosilane as a pro-nucleophile or a base-embedding electrophile will depend on several factors. The methods are obviously complementary, as almost always is the case in organic synthesis. Firstly, there will be many instances in which both can be tested. The base-embedding electrophile strategy makes sense in cases where an organosilane is not easily synthesized and/or is unstable, or when one simply wants to skip the preparation of an organosilane, an issue that is most relevant to complex molecule synthesis or process chemistry. On one hand, BEEs can be changed to tune basicity. All reactions shown herein were promoted by only five BEEs; one possible approach to employing less acidic substrates is perhaps to develop new BEE structures. On the other hand, the same BEE can give rise to different anionic nucleophiles, making assessment of scope more practical and faster. A BEE does not necessarily need to be silicon-based; previous activation of stannanes with bromide have been reported,^75^ and we envision BEEs with other atoms in the p block of the periodic table, most intuitively B-, Al-, or Ge-containing. We also wonder whether a BEE can be made to generate extremely reactive and elusive ion pairs, such as carbon-based alkyl or aryl anions balanced by organic cations. Such anions could further extend the p*K*_a_ limit of pro-nucleophiles, enabling elusive catalytic deprotonations.

In their seminal publication in 1977 (Fig. 1),^16^ Noyori and Kuwajima made the following remark, when referring to another seminal work by Mukaiyama published in 1974^6^: “In view of such mildness of the conditions, the present aldol reaction, complementary to the previously discovered TiCl_4_-promoted reaction, provides a new tool in organic synthesis.” The statement turned out to be correct and the past ten years have demonstrated that there is room for several developments. For example, while the mildness of the Mukaiyama aldol reaction, with its many possibilities for stereoselectivity induction based on chiral Lewis acids, has proved difficult to overcome, this review shows that other reactions have been explored. Many more are yet to be discovered, and we attest that our laboratory is currently developing reactions that we believe are worth the effort. In this context, providing more robust and modular chiral enantioenriched fluoride (or other anion) pre-catalysts should greatly impact the field. In principle, one could also evaluate the use of pre-catalysts with substoichiometric amounts of sacrificial reagents for generation of more active catalysts or to enter the catalytic cycle more efficiently and controllably. Herein, we report and present ideas in the context of small-molecule organic synthesis, yet the concepts are likely to have implications in polymer synthesis and materials.

Lastly, the catalysis displayed herein is distinct in the way the catalyst is regenerated. Catalyst turnover takes place by reaction of an anionic nucleophile with an electrophile, disentangling access to reactive anionic Lewis bases from Brønsted acid–base constraints. This is most relevant to substitution processes, but it also imparts addition, and perhaps in the future, elimination reactions.

## Author contributions

P. H. S. P. conceptualized the review. All authors contributed to writing, reviewing and editing the manuscript.

## Conflicts of interest

There are no conflicts to declare.

## Data Availability

No primary research results, software or code have been included, and no new data were generated or analysed as part of this review.
